# Attention Deficit Hyperactivity Disorder in Australian Adults: Prevalence, Persistence, Conduct Problems and Disadvantage

**DOI:** 10.1371/journal.pone.0047404

**Published:** 2012-10-10

**Authors:** Jane L. Ebejer, Sarah E. Medland, Julius van der Werf, Cedric Gondro, Anjali K. Henders, Michael Lynskey, Nicholas G. Martin, David L. Duffy

**Affiliations:** 1 School Of Rural Science and Agriculture, University of New England, Armidale, New South Wales, Australia; 2 Genetic Epidemiology, Queensland Institute of Medical Research, Brisbane, Queensland, Australia; 3 Department of Psychiatry, Washington University School of Medicine, St Louis, Missouri, United States of America; University of Wuerzburg, Germany

## Abstract

**Background:**

The Prevalence and persistence of ADHD have not been described in young Australian adults and few studies have examined how conduct problems (CP) are associated with ADHD for this age group. We estimate lifetime and adult prevalence and persistence rates for three categories of ADHD for 3795 Australian adults, and indicate how career, health and childhood risk factors differ for people with ADHD symptoms and ADHD symptoms plus CP.

**Methodology:**

Trained interviewers collected participant experience of ADHD, CP, education, employment, childhood experience, relationship and health variables. Three diagnostic definitions of ADHD used were *(i)* full DSM-IV criteria; *(ii)* excluding the age 7 onset criterion (*no age criterion*); *(iii)* participant experienced difficulties due to ADHD symptoms (*problem symptoms*).

**Results:**

Prevalence rates in adulthood were 1.1%, 2.3% and 2.7% for each categorization respectively. Persistence of ADHD from childhood averaged across gender was 55.3% for full criteria, 50.3% with no age criterion and 40.2% for problem symptoms. ADHD symptoms were associated with parental conflict, poor health, being sexually assaulted during childhood, lower education, income loss and higher unemployment. The lifetime prevalence of conduct problems for adults with ADHD was 57.8% and 6.9% for adults without ADHD. The greatest disadvantage was experienced by participants with ADHD plus CP.

**Conclusion:**

The persistence of ADHD into adulthood was greatest for participants meeting full diagnostic criteria and inattention was associated with the greatest loss of income and disadvantage. The disadvantage associated with conduct problems differed in severity and was relevant for a high proportion of adults with ADHD. Women but not men with ADHD reported more childhood adversity, possibly indicating varied etiology and treatment needs. The impact and treatment needs of adults with ADHD and CP and the report of sexual assault during childhood by women and men with ADHD also deserve further study.

## Introduction

Attention deficit hyperactivity disorder (ADHD) describes a range of behaviours characterized by forgetfulness, carelessness, inattention, disorganization, distractedness, fidgetiness and impulsivity possibly associated with inhibitory deficits [1], [2]. Onset is classically in early childhood with prevalence at age 7 estimated to range between 3% and 7% [3]. Approximately 70% of the variation in ADHD symptoms in childhood is accounted for by genetic effects [4], [5], but only 30% in a sample of adults [6]. Experiences specific to an individual appear to account for a greater degree of the variation of ADHD symptoms in adulthood. Identification of environments more strongly associated with ADHD could indicate how and why symptoms vary under certain conditions [7].

Known risk factors for childhood symptoms include low birth weight [8], negative attention from parents [9], physical and emotional neglect [10]. ADHD symptoms have a negative impact on affected individuals, on their families [11] and within our communities [12]. Children affected by symptoms have increased difficulties with reading [13], motor performance [14], emotional regulation and social interaction [15]. They are also at increased risk for substance use, conduct problems, mood disorders and anxiety [16], [17]. As adults this extends to include lower educational attainment, a greater likelihood of multiple marriages, lost productivity at work [18], [19], experiencing a second disorder [20] and an increased likelihood of unemployment [21].

The expression of ADHD symptoms in adulthood vary from the childhood criteria listed in the DSM-IV [22], additionally the number of symptoms declines but disability is still evident. Kooij et al. [23] found four or more symptoms of either inattention or hyperactive-impulsivity were associated with higher levels of disability for 1813 adults aged from 18 to 75. Das et al. [17] measured ADHD as a trait in 2091 adults aged 47 to 54 indicating higher trait scores but not necessarily in the clinical range were associated with financial stress, reduced employment, marital difficulties and lower subjective well-being The persistence of ADHD into adulthood has been reported to range from 4% for full DSM-IV diagnostic criteria [24], to 90% when persistence of impairment is considered as a more relevant criterion [25]. Approximately 6% of adult populations may be disadvantaged by ADHD symptoms [20], [26].

Higher levels of disadvantage are associated with ADHD when conduct problems are also present [27]–[29]. It is unclear whether or not this is due to the conduct problems or the environment and little work has examined the impact of conduct problems on ADHD symptoms and associated disadvantage in adulthood.

We address several important questions. *(i)* What is the sample prevalence rate for lifetime and adult ADHD in the Australian population? *(ii)* What is the rate at which symptoms persist into adulthood? *(iii)* Are adults reporting ADHD symptoms more intensely exposed to childhood risk factors? *(iv)* How are Australian adults disadvantaged by ADHD symptoms? And *(v)* what is the impact of conduct problems on the disadvantage reported by adults with ADHD?

## Methods

### Participants

**Figure 1:**
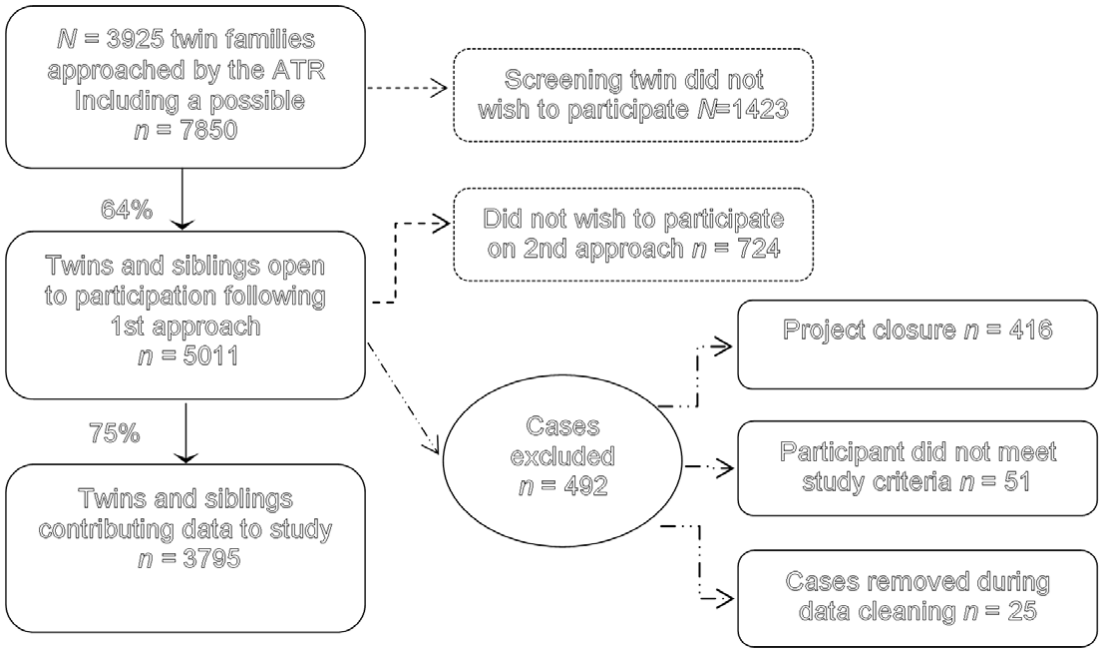
Flow chart indicating representing the proportion of participants to possible participants. Legend: The family participation rate was 63.7% and the rate for individuals was 48.3%.

Participants were monozygotic (MZ) and dizygotic (DZ) twins and their siblings in total 1369 men and 2426 women, recruited through the National Health and Medical Research Council Twin Registry (ATR). The ATR was established to collect data from twins and their families for medical and scientific research. For this study, ATR members born between 1972 and 1979 were invited by mail to participate in a project examining the genetics of cannabis use and mental health. If a twin indicated a willingness to participate they were sent a pack providing detailed information about the study and a consent form. The twins who then wished to be involved returned their written consent to the ATR along with their agreement to possible contact with additional family members. Recruitment was multi-stage and of the 3925 initial recruitment letters sent to families at least one twin from 2405 families responded giving a family participation rate of 63.7%. The individual participation rate described in Figure 1 was 48.3% and Table 1 displays descriptive statistics for study participants. Twins have previously been found to have a higher incidence of ADHD than singletons [30] this did not appear to be the case in our sample [*x* ^2^(1) = 0.03, *p* = .87].

**Table 1:** Participant Mean Age at Assessment, Sex and Zygosity.

Zygosity & Sex	Individuals	CompletePairs	Age(SD)
MZ female	981	400	31.8 (2.6)
MZ male	483	171	31.7 (2.4)
DZ female	742	302	31.7 (2.5)
DZ male	377	119	31.8 (2.4)
DZ opposite sex	740	225	32.1 (2.5)
Non-twin sisters	267	–	34.2 (5.2)
Non-twin brothers	205	–	33.6 (5.0)
Total	3795		

*Note:* The age within the final sample ranged from 21 to 49.

#### Ethics statement

Ethics approval for this study was provided by the Queensland Institute of Medical, Research Human Research Ethics Committee, ATR ethics and Washington University School of Medicine Human Research Protection Office. Written and informed consent was provided by all study participants.

### Measures

Data were collected using a combination of computer assisted telephone interview (CATI) administered by trained research interviewers and either an online or paper version of a Health and Lifestyle Personality Questionnaire (HLPQ). The CATI was structured to collect self-reported sociodemographic variables; family environment during childhood, school grades, educational attainment, emotional and physical well-being, marital status, income level, employment, sexual assault prior to 18 and data relevant to psychiatric classification. The Semi-Structured Assessment for the Genetics of alcoholism (SSAGA; Bucholz et al., 1994) [31] which is based on diagnostic criteria of the DSM-III-R and DSM-IV, collected data on psychiatric classifications. This included 34-items addressing ADHD diagnostic criteria and 109-items specific to conduct problems. For this study we selected respondent data on the ADHD, conduct problems and sociodemographic variables.

#### Sociodemographic variables

The sociodemographic variables were divided into three categories; those related to *career path*, childhood circumstances associated with poor outcomes (*childhood risk factors*) and *health and lifestyle* variables. The career related variables included educational attainment and within the Australian school system there were two valid exit points at which students could gain recognition of achievement; either year 10 or years 10 and 12, but students may have chosen to leave at any time up to year 12. Scholastic and trade qualifications could also be attained through a college of Techincal and Further Education (TAFE). Level of educational attainment was coded as *1≤ year 10, 2 = year 11 or 12, 3 = TAFE* and *4≥ degree.* School grades during primary and high schools were coded as *1< average, 2 = average* and 3*> average* and weekly income was condensed into categories; *1≤ $481, 2 = $482−$769, 3 = $770−$1442,* and *4≥ $1442.* Occupation was also coded into four categories; *1 = not working, 2 = student, 3 = working* and *4 = homemaker*.

Childhood risk was primarily measured by circumstances within the family during childhood between the ages of 6 and 13. The families socio-economic status was coded as *1< average, 2 = average* and *3> average.* Conflict with parents, parental arguing, parental tension and drinking were scaled as; *1 = never or rarely*, *2 = sometimes* and *3 = often*, parental rules were coded as *1 = inconsistent* and *2 = consistent.* Being raised by both parents to the age of sixteen was coded as *1 = no* and *2 = yes* and experiencing a sexual assault during childhood was coded as *1 = no, 2 = once, 3 = more than once.* Health and lifestyle variables included marital status which was coded as; *1 = unmarried, 2 = prior marriage* and *3 = married*, physical and emotional health were measured as *1 = poor, 2 = fair, 3 = good or excellent*. The continuous variables of days ill per year (*M* = 7.3, *sd* = 23.0) and birth weight (*M* = 2703.0, *SD = *684.6) were coded as *1 = 0−10, 2 = 11−30*, *3>30* and *1<2333 g* and *2≥2333 g* respectively. The report of problem drinking was coded as *1 = no* and *2 = yes.* The low birth weight represented in analyses (2333 g) was at the 10^th^ percentile for twins [32]. Within twin pairs, lower birth weight was not associated with inattentive (*p = *.52) or hyperactive-impulsivity symptoms (*p = *.43).

#### Attention-deficit/hyperactivity disorder

Participants’ report of ADHD symptoms were used to create three diagnostic definitions of ADHD and each was divided into *lifetime risk* and *current* diagnoses. First, full DSM-IV diagnostic criteria for primary inattention, hyperactive-impulsivity and combined ADHD: *(A)* over a 6-month period the experience of six of nine possible inattentive or hyperactive-impulsive symptoms for each subtype respectively and at least 6 inattentive and 6 hyperactive-impulsive symptoms for the combined subtype; *(B)* symptom onset by the age of 7 years; *(C)* symptoms experienced across two life arenas - either home and school for childhood retrospective report and work for the report of continuing symptoms, home and socializing or school/work and socializing; *(D)* problems resulted from ADHD symptoms; and *(E)* exclusion of autism and Asperger’s disorder. The second ADHD measure included all criteria with the exception of *B* – the age of symptom onset was left open. The third measure represented participants who met only criteria C (*impairment across two settings*) and/or D (*impairment was clinically relevant).* The report ADHD symptoms caused problems in a persons’ life was our measure of clinical relevance. Participant report of symptoms within the previous 12 months differentiated lifetime risk from current diagnoses.

#### Conduct problems

Data on symptoms of conduct disorder were collected if participants reported they occurred prior to the age of 18. A symptom count of conduct problems was entered as a covariate in regression analyses and the following DSM-IV criteria were used for diagnosing ADHD with co-morbid conduct problems: (A) three symptoms across three behaviours including – *i*. aggression to people and animals, *ii*. destruction of property, *iii*. deceitfulness or theft and/or serious violation of rules; (B) symptoms have caused problems in social academic or occupational functioning; and (C) a diagnosis of anti-social personality disorder was not applicable. Antisocial personality disorder (ASPD) was diagnosed according to the listed criteria A and B, with the inclusion that; by the age of 15 three symptoms had been experienced within 12 months.

### Interview Procedures

Once a twin initiated contact with the ATR an interview time was arranged. The CATI response booklet was sent to the respondent along with information guiding them to the online HLPQ and they were invited to complete the HLPQ prior to the CATI. During the interview respondents, referred to the CATI response booklet when asked by interviewers to indicate their responses. All interviews were recorded and reviewed by editors to ensure consistency across the duration of the study and interviewers.

## Analyses

### Missing Values

Across variables there were little missing data. The missingness in inattention and hyperactivity-impulsivity items was 2.1%, conduct problems had 3.1% to 4.1%. The career, childhood risk and health related variables had 1.0%, 0.5% and 0.2% missing values respectively, with the exception of *alcohol problem* and *days ill per year*. These two variables were only available for a reduced subset of participants due to a change in protocol (data were available for 2681 and 2678 individuals respectively). All analyses were run with available data.

### Prevalence and Rates of Persistence

Persistence of ADHD into adulthood was calculated with survival analysis using the age of participants at symptom onset, the age at which symptoms were last experienced and whether or not symptoms were remitting. This method takes into consideration the entry and exit of participants to and from the pool of individuals affected by ADHD across time. So participants with a late symptom onset were excluded from the final rate because they did not contribute to the estimate of prevalence before the first remission occurred. Three prevalence rates were calculated corresponding to the three sets of criteria used to measure symptoms of ADHD. Each of the prevalence estimates was standardized to the gender and education distributions of the Australian population to account for the higher prevalence of ADHD in men and the association between ADHD and lower educational attainment.

### Regression of ADHD Symptoms Onto Sociodemographic Variables

Ordinal logistic regression (OLR) was used to examine the relationships between ADHD symptoms, conduct problems and sociodemographic variables. Analyses were conducted for the full sample, and differentiated by gender when a significant variable by gender interaction was indicated. Inattentive and hyperactive-impulsive symptom counts were modeled as the outcome variables and all regressions were run twice, the second run included *conduct problem symptom count* as a covariate. The results of these analyses were weighted to account for the similarity of response among related participants. Weighting was calculated using the averaged correlation between twins within MZ and DZ pairs for ADHD as a proportion of the total variance [33] which was 1.83. The standard error for each analysis was multiplied by this value to increase the variance to what would be expected within an independent sample.

## Results

### The Persistence and Prevalence of ADHD in Adulthood

#### DSM-IV ADHD full criteria

**Figure 2:**
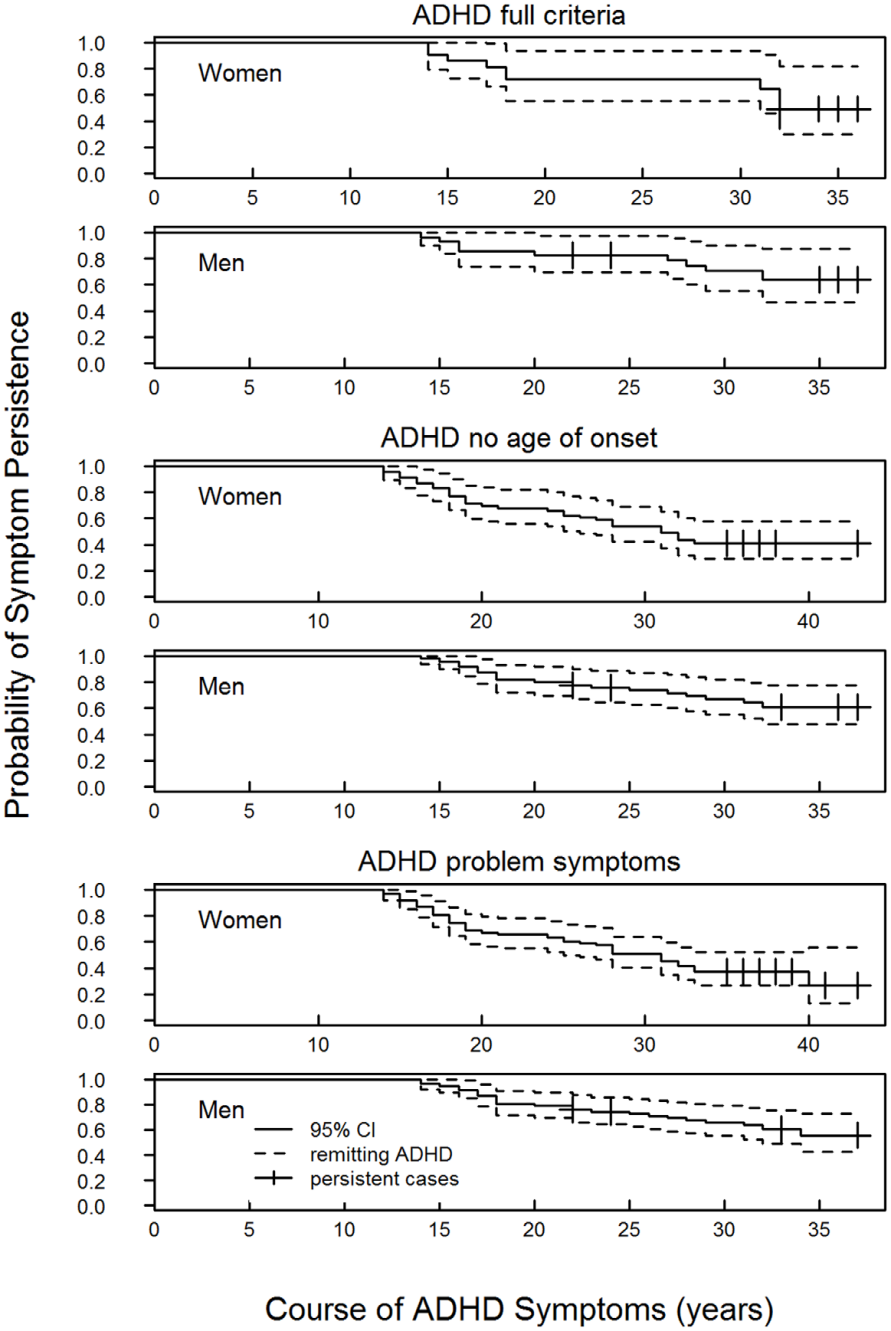
Probability of ADHD persistence and pattern of remission for each ADHD categorisation by gender.

The lifetime prevalence of ADHD was 0.9% for women (21/2406) and 2. 1% for men (28/1341). There were 0.5% of women (12/2415) and 1.4% of men (19/1350) with current ADHD, standardized and raw prevalence estimates across education and gender categories are presented in Table 2. The rate at which symptoms persisted into adulthood was 47.6% (27.9% –81.3%) and 63.0% (45.5% –87.2%) for women and men respectively as presented in Figure 2. The prevalence [*x* ^2^(1) = 7.22, *p* = .007] but not persistence of symptoms [*x* ^2^(1) = 0.22, *p* = .64] was higher for men. Prevalence rates were not associated with birth year which was represented in quartiles *x* ^2^(3) = 0.64, *p = *.89, possibly due to the restricted age of the sample. The prevalence of antisocial personality disorder within this ADHD categorization was 35.5% (*SD* = 6.3%) compared to 3.4% (*SD* = 0.1%) in the full sample. MZ twins, DZ twins and singletons did not differ significantly in prevalence of symptoms and diagnoses [approximately *x* ^2^(25) = 30.0, *p* = .22 for each comparison].

**Table 2:** Raw Prevalence of ADHD in Adulthood By Sex and Educational Attainment With Adjusted Totals.

Highest Levelof Education	Frequency(n)	DSM-IVADHD	Std.Error	ADHD noAgeCriterion	Std.Error	ProblemSymptoms	Std.Error	Gender ByEducationWeighting
*Females*								
Year 10≤	142	1.41	0.99	4.23	1.69	4.93	1.82	.12
Year 11	95	1.06	1.06	1.06	1.06	1.06	1.06	.04
Year 12	338	0.00	0.00	0.89	0.51	0.89	0.51	.11
TAFE	651	0.31	0.22	0.92	0.37	1.08	0.41	.06
Undergraduate	708	0.85	0.35	1.41	0.44	1.55	0.46	.14
Post graduate	525	0.19	0.19	0.38	0.27	0.57	0.33	.03
*Adjusted*								
* Sub-total*	2459	0.35	0.14	0.89	0.17	1.01	0.18	.50
*Males*								
Year 10≤	117	0.85	0.85	3.42	1.68	5.13	2.04	.11
Year 11	76	6.58	2.84	6.58	2.84	6.58	2.84	.03
Year 12	194	2.07	1.03	3.63	1.35	4.15	1.43	.10
TAFE	443	1.13	0.50	2.26	0.71	2.71	0.77	.11
Undergraduate	352	0.86	0.49	1.43	0.63	1.71	0.69	.11
Post graduate	233	043	0.43	0.43	0.43	0.86	0.60	.03
*Adjusted*								
* Sub-total*	1415	0.77	0.17	1.38	0.24	1.77	0.27	.50
Total	3874	1.11^a^	0.20	2.34^a^	0.30	2.72^a^	0.32	1.00

*Note*. ^a^Total prevalence was calculated by multiplying the prevalence of ADHD within each gender and educational group (row) by the weight in the final column. Diagnostic categories are nested so participants meeting criteria for more restrictive categories are also included in the broader categorizations.

#### DSM-IV ADHD with no age of onset criterion

The lifetime prevalence of ADHD when only age of onset criterion was dropped was 3.8% for men (50/1319) and 2.4% for women (58/2369). Symptoms were reported as current by 1.2% women (29/2398) and 2.4% men (32/1337). Since not all participants had experienced symptoms by the age of the first remission (which was 14), we adjusted for both left and right censoring to estimate a persistence rate of 60.2% (46.9% –77.2%) for men and 40.3% (28.2% –57.4%) for women. Again the prevalence but not persistence of symptoms was significantly higher for men *x* ^2^(1) = 6.75, *p = *.009 and *x* ^2^(1) = 1.83, *p* = .18 respectively and there was no difference in ADHD prevalence across birth year *x* ^2^(1) = 1.03, *p = *.79. There were 26.7% (*SD* = 3.5%) of these participants meeting criteria for antisocial personality disorder.

#### Measure of persisting problem ADHD symptoms

The problem symptoms category represented participants meeting DSM-IV ADHD criterion *C impairment is evident in two or more settings* and/or *D impairment is clinically relevant*. It was not a requirement that these participants met criterion *A* (*six inattentive or hyperactive-impulsive symptoms*) so the lifetime prevalence of problematic ADHD symptoms–3.1% for women (74/2352) and 4.8% for men (63/130) – was higher than the previous two categories. There were 2.9% and 1.4% of men and women respectively within this category, reporting current symptoms and this gender difference was significant *x* ^2^(1) = 9.77, *p = *.002. Persistence of symptoms into adulthood was 55.7% (42.5% –73.1%) for men and 24.6% (10.3% –58.5%) for women, this difference reached significance *x* ^2^(1) = 3.73, *p = *.05. Again there was some censoring due to a later age of symptom onset for participants with problem symptoms and all had experienced at least six or more ADHD symptoms, which was the criterion for asking whether or not symptoms were experienced as problematic. The prevalence of anti-social personality disorder within this group was 23.9% (*SD* = 2.8%).

### Career Related Outcomes, Inattention, Hyperactive-Impulsivity and Conduct Problems

Ordinal Logistic Regression of career related variables onto ADHD symptoms was conducted for the full sample and results are provided in Table 3. Participants with higher levels of inattention with and without conduct problems attained lower levels of education than participants with none or few symptoms (*p*<.01 and *p*<.05 respectively). Men and women reporting low grades in primary and high schools also reported more symptoms of inattention (*p*<.001) and hyperactivity-impulsivity (*p*<.05). The odds ratios indicated this relationship was strongest when conduct problems were also present. Both women and men reporting inattentive symptoms earned less money each week than participants with fewer or no inattentive symptoms. Participants with higher levels of inattention were more likely to be unemployed (*p*<.05) and the likelihood increased when they had also experienced conduct problems (*p<*.001).

**Table 3:** Odds Ratio (CI) Of ADHD Symptoms With and Without CP Within Career Categories.

		ADHD	ADHD	ADHD + CP	ADHD + CP
CareerVariables	Frequency(*n*)	*Inattention*	*Hyperactive-* *impulsive*	*Inattention*	*Hyperactive-* *Impulsive*
*Education*					
≤ Year 10	259	(1)	(1)	(1)	(1)
Year 11 or 12	701	0.86	0.90	0.64	0.69
		(0.41–1.81)	(0.42–1.92)	(0.32–1.31)	(0.33–1.42)
TAFE	1093	0.70	0.83	0.49*	0.59
		(0.34–1.44)	(0.40–1.73)	(0.24–0.96)	(0.30–1.19)
Degree	1817	0.47*	0.73	0.28**	0.44*
		(0.23–0.98)	(0.35–1.49)	(0.14–0.56)	(0.22–0.86)
*Primary School*					
<Average	174	(1)	(1)	(1)	(1)
Average	1797	0.13***	0.39*	0.11***	0.32***
		(0.07–0.24)	(0.19–0.78)	(0.06–0.21)	(0.16–0.62)
>Average	1817	0.10***	0.39*	0.08***	0.29***
		(0.05–0.19)	(0.19–0.80)	(0.04–0.15)	(0.15–0.58)
*High School*					
<Average	226	(1)	(1)	(1)	(1)
Average	1943	0.20***	0.39*	0.14***	0.32***
		(0.11–0.37)	(0.19–0.78)	(0.08–0.25)	(0.17–0.59)
>Average	1622	0.15***	0.39*	0.09***	0.31***
		(0.08–0.28)	(0.19–0.80)	(0.05–0.16)	(0.17–0.58)
*Income*					
≤$481	1051	(1)	(1)	(1)	(1)
$482*−*$769	636	0.92	0.73	1.00	0.80
		(0.52–1.64)	(0.41–1.33)	(0.57–1.73)	(0.45–1.42)
$770*−*$1442	1362	0.59*	0.73	0.61*	0.74
		(0.36–0.98)	(0.45–1.17)	(0.37–1.00)	(0.46–1.18)
≥ $1443	789	0.50*	0.83	0.53*	0.85
		(0.27–0.94)	(0.48–1.43)	(0.29–0.98)	(0.50–1.44)
*Occupation*					
Not Working	101	(1)	(1)	(1)	(1)
Student	96	0.43	1.03	0.31	0.68
		(0.11–1.79)	(0.26–4.12)	(0.08–1.22)	(0.18–2.54)
Working	3201	0.29*	0.57	0.21***	0.39*
		(0.12–0.69)	(0.22–1.50)	(0.09–0.47)	(0.16–0.96)
Homemaker	472	0.35*	0.66	0.22**	0.40
		(0.12–0.96)	(0.22–1.97)	(0.08–0.59)	(0.14–1.13)

Note: * p≤.05, ** p≤.10, *** p≤.001. CI = confidence intervals.

### Childhood Risk Factors, Inattention, Hyperactive-Impulsivity and Conduct Problems

As summarized in Table 4 symptoms of inattention and hyperactive-impulsivity were predicted by low socio-economic status during childhood in association with conduct problems (*p*<.05). A relationship between hyperactive-impulsivity and being raised in a one-parent household was predicted by comorbid conduct problems (*p*<.05) but this relationship was suggested for inattention with conduct problems and hyperactive-impulsivity only. There was a strong relationship between childhood sexual assault, inattentive and hyperactivity-impulsive symptoms (*p*<.001 and *p*<.01 respectively) whether or not conduct symptoms were also experienced. There were significant gender interactions; conflict with parents, parental arguing, parental tension and parental rules predicted more symptoms of ADHD for women but not men. This relationship was strongest for women reporting conflict with their parents during childhood (*p*<.001), but also apparent with the report of more parental tension (*p<*.05). Inconsistent parental rules predicted inattention with and without conduct problems for women but not for men (*p*<.01 and *p*<.05, respectively). Parental arguing predicted inattention and hyperactivity-impulsivity for women but only when they occurred with conduct problems (*p*<.05). There was no systematic relationship between parental drinking and symptoms of ADHD. Results for gender specific analyses are available from the first author on request.

**Table 4:** Odds Ratio (CI) Of ADHD Symptoms With and Without CP For Childhood Risk Factors.

		ADHD	ADHD	ADHD + CP	ADHD + CP
Childhood RiskFactors	Frequency(n)	*Inattention*	*Hyperactive-* *impulsive*	*Inattention*	*Hyperactive-* *Impulsive*
*CSA*					
No	3463	(1)	(1)	(1)	(1)
Once	128	1.61	1.18	1.87	1.36
		(0.62–4.16)	(0.44–3.16)	(0.75–4.66)	(0.52–3.54)
>Once	199	2.87***	2.58**	4.30***	3.61***
		(1.52–5.42)	(1.37–4.86)	(2.37–7.79)	(1.97–6.59)
*Childhood SES*					
< Average	519	(1)	(1)	(1)	(1)
Average	2721	0.68	0.68	0.54*	0.58*
		(0.40–.13)	(0.40–11.13)	(0.32–.90)	(0.35–0.96)
>Average	626	1.00	1.00	0.73	0.92
		(0.54–.87)	(0.54–1.87)	(0.38–.39)	(0.50–1.69)
*With both parents*					
No	684	(1)	(1)	(1)	(1)
Yes	3188	0.87	0.64	0.68	0.54*
		(0.53–.43)	(0.41–.01)	(0.43–.10)	(0.35–0.83)
*Conflict w Parents*					
Never/Rarely	2008	(1)	(1)	(1)	(1)
Sometimes	1397	1.48	1.26	1.73*	1.46
		(0.93–2.36)	(0.82–1.95)	(1.10–2.72)	(0.96–2.24)
Often	465	2.61***	2.46***	3.80***	3.41***
		(1.49–4.57)	1.46–4.16)	(2.23–6.46)	(2.07–5.62)
*Parental arguing*					
Never/Rarely	2570	(1)	(1)	(1)	(1)
Sometimes	871	1.47	1.31	1.54	1.37
		(0.92–2.36)	(0.83–2.06)	(0.97–2.44)	(0.88–2.13)
Often	416	1.62	1.48	1.96*	1.73*
		(0.90–2.93)	(0.83–2.61	(1.11–3.46)	(1.00–2.99)
*Parental Tension*					
None/A little	2822	(1)	(1)	(1)	(1)
Some	651	1.47	1.07	1.62	1.18
		(0.88–2.45)	(0.64–1.78)	(0.98–2.66)	(0.72–1.95)
A lot	397	2.17*	1.76*	2.60***	2.02*
		(1.24–3.78)	(1.02–3.03)	(1.52–4.44)	(1.19–3.43)
*Parental Rules*					
Inconsistent	366	(1)	(1)	(1)	(1)
Consistent	3500	0.54*	0.75	0.44**	0.60
		(0.31–0.96)	(0.42–1.35)	(0.26–0.75)	(0.35–1.05)
*Parental Drinking*					
Never/Rarely	1259	(1)	(1)	(1)	(1)
Sometimes	1404	0.63	0.83	0.59*	0.79
		(0.37–1.06)	(0.52–1.33)	(0.35–0.98)	(0.50–1.24)
Often	1207	1.05	0.91	1.16	1.01
		(0.66–1.69)	(0.57–1.45)	(0.73–1.83)	(0.64–1.59)

*Note:* These variables represent participant retrospective report of home life from the age of 6 to 13. *p≤.05, **p≤.10, ***p≤.001. CSA = sexual assault during childhood.

### Health Related Outcomes, Inattention, Hyperactive-Impulsivity and Conduct Problems

Poor physical and emotional health were predicted by higher levels of inattention and hyperactivity with and without conduct problems (*p*<.05 to *p*<.001; Table 5). There were significant interactions between gender and ADHD symptoms for days ill per year and birth weight. Women with symptoms of inattention on their own and with conduct problems, reported being ill for a relatively high number of days each year (*p*<.001). However there was no significant effect of birth weight when gender was differentiated, though females generally have a lower birth weight than males [32]. The relationship between ADHD symptoms and a reduced likelihood of marriage only reached significance when participants also reported conduct problems (*p*<.05).

**Table 5:** Odds Ratio (CI) Of ADHD Symptoms With and Without CP Within Health Related Categories.

		ADHD	ADHD	ADHD + CP	ADHD + CP
Health & LifestyleVariables	Frequency(n)	*Inattention*	*Hyperactive-* *impulsive*	*Inattention*	*Hyperactive-* *impulsive*
*Days III year*					
0–10	3073	(1)	(1)	(1)	(1)
11–30	317	2.40*	1.71	2.71***	1.92*
		(1.29–4.44)	(0.93–3.14)	(1.51–4.86)	(1.07–3.43)
>30	98	3.85**	1.91	4.78***	2.47
		(1.59–9.31)	(0.72–5.06)	(2.06–11.06)	(0.98–6.22)
*Birth Weight*					
<2333 g	451	(1)	(1)	(1)	(1)
≥2333 g	1181	0.74	0.85	0.83	0.90
		(0.42–1.33)	(0.52–1.41)	(0.40–1.72)	(0.48–1.70)
*Physical health*					
Poor	110	(1)	(1)	(1)	(1)
Fair	668	0.64	0.50	0.61	0.49
		(0.24–1.67)	(0.19–1.27)	(0.24–1.53)	(0.20–1.20)
≥ Good	3090	0.32*	0.35*	0.27**	0.30*
		(0.13–0.80)	(0.15–0.84)	(0.11–0.64)	(0.13–0.69)
*Emotional health*					
Poor	92	(1)	(1)	(1)	(1)
Fair	582	0.43	0.68	0.35*	0.54
		(0.18–1.03)	(0.27–1.74)	(0.15–0.81)	(0.22–1.35)
≥ Good	3194	0.10***	0.26**	0.08***	0.19***
		(0.04–0.23)	(0.11–0.64)	(0.03–0.17)	(0.08–0.46)
*Close friends*					
No	132	(1)	(1)	(1)	(1)
Yes	3736	0.46	0.51	0.38*	0.45
		(0.20–1.07)	(0.22–1.19)	(0.17–0.85)	(0.20–1.00)
*Marital status*					
Unmarried	1593	(1)	(1)	(1)	(1)
Prior marriage	201	1.20	1.04	1.09	0.99
		(0.52–2.80)	(0.46–2.33)	(0.48–2.46)	(0.45–2.18)
Married	2080	0.74	0.69	0.62*	0.60*
		(0.48–1.13)	(0.46–1.02)	(0.41–0.94)	(0.41–0.88)
*Alcohol problem*					
No	3427	(1)	(1)	(1)	(1)
Yes	103	1.37	1.75	3.55**	3.96***
		(0.52–3.56)	(0.71–4.28)	(1.52–8.30)	(1.77–8.84)

Note: *p≤.05, **p≤.10, ***p≤.001. CI = confidence intervals

## Discussion

The average prevalence of adult ADHD within the sample was 1.1% using full criteria, 2.3% when the age of onset criterion was relaxed and 2.7% for problem symptoms (criterion C and/or D). Past prevalence estimates of ADHD within adult populations range between 1.0% found in Holland [23] and approximately 10.0% in South America [26]. In support of our findings, a study of older Australians indicated 1.0% of their sample had high levels of ADHD symptoms [17]. However our estimate for a full diagnosis is most similar to the results of Kooij and colleagues their rate of persistence was determined by the experience of symptoms within the previous 6-months in contrast to the 12-months we used in this study and their age of onset extended to 8-years old. A cross-national study indicating the prevalence of DSM-IV ADHD in adults was 3.4% [20] also used retrospective self-report and indicated our estimates were comparatively low. Within their study of adults aged from 18 to 44, Fayyad and colleagues also found men had a higher prevalence than women as was found in this study. In contrast Kooij and colleagues found no sex difference in their prevalence of DSM-IV diagnosed ADHD and a higher rate for women when using a four rather than the six symptom criterion A, the age of their participants ranged from 18 to 75. Similarly Das and colleagues [17] found no sex differences in the prevalence of ADHD in their older sample. The differences in the prevalence of adult ADHD across studies suggests variation in the expression of ADHD could be related to developmental change [22] cultural differences across populations and/or differences in methodology across studies [24] as has previously been suggested.

The persistence of ADHD into adulthood occurred for 24.6% to 63.0% of ADHD affected women and men across ADHD categorizations. The highest degree of persistence occurred for adults meeting full DSM-IV criteria, symptom severity has previously been found to predict symptom persistence [34]. The persistence found in a study by Kessler and others was also similar to ours (28.0% to 49.3%) and corresponded to increasingly restrictive diagnostic criteria. Persistence and prevalence rates of ADHD vary with the diagnostic criteria used to define symptoms [24]. Additionally ADHD diagnoses have been found to vary across raters and scales [35] so our estimates cannot be considered as true but relative to the study design we used.

Our finding that childhood risk factors did predict higher levels of ADHD symptoms supports previous research indicating an association between childhood adversity and retrospective report of ADHD [34]. We extended this work and examined sex differences. More conflict with parents, more parental arguing, inconsistent parental rules and more parental tension predicted more inattentive and hyperactive-impulsive symptoms for women but not for men. It may be that females and males vary in their perception of parental behaviour [36], or boys and girls are treated differently by their parents. Children with ADHD, most particularly inattention more frequently report lack of supervision, neglect, physical and sexual abuse than children without ADHD symptoms [37]. Sexual assault during childhood predicted higher symptom count of inattentive and hyperactive-impulsive symptoms. ADHD is not a natural sequela of child sexual assault but higher levels of conduct problems, depression, anxiety, alcoholism and panic disorder have previously been found for adults reporting childhood sexual abuse [38].

The disadvantage adults with ADHD symptoms reported included lower educational attainment, loss of income, increased unemployment, poor physical health, more days ill each year, reduced likelihood of marriage and fewer close friends. The disadvantage associated with comorbid conduct problems did not appear to differ qualitatively from that associated with ADHD symptoms but was more severe. The report of conduct problems was uniquely associated with being raised by one parent only, lower socio-economic status, more conflict with parents and problem drinking though these trends were evident for participants with ADHD symptoms only. The lifetime prevalence of conduct problems for participants with no history of ADHD was 6.9%. For participants with a history of ADHD this rate increased to 67.9% and the high incidence of antisocial personality disorder indicated ongoing and severe difficulties for ADHD affected adults.

There were several limitations that influence the interpretation of our findings. The survival analysis was based on the report of the first and last experience of symptoms. Even though a participant may have met full diagnostic criteria the variation of these symptoms across time could not be measured precisely. Further the use of a CATI rather than a clinician appraisal to diagnose ADHD may have resulted in a loss of rapport only possible in a face-to-face interview, and influenced our findings. Our prevalence rate of adult ADHD was comparatively low to previous estimates and the adjustment made for gender and education could not fully account for this difference and we were unable to estimate the effect due to methodological variation in ADHD diagnoses. The degree to which missing values were random could not be defined. But, participants returning the HLPQ and not completing the CATI did not appear to differ from full participants in rates of substance use, autism, Asperger’s syndrome or ADHD (data are not shown).

In conclusion our findings indicate ADHD in adults occurs for a high proportion of people who report symptoms during their earlier years. These symptoms are associated with income loss for men and women and according to our criteria affect approximately 250 000 to 750 000 Australians at a conservative estimate. Identification of disadvantage associated with subclinical ADHD symptoms and associated conduct problems in adults and a possible need for treatment will help to alleviate personal, familial and community issues resulting from the symptoms reported by these adults. Further research into the increased number of ADHD symptoms associated with childhood adversity for women and for women and men also experiencing a sexual assault during childhood could indicate ways in which symptoms and etiology varies across gender and how treatments may also need to vary.

## References

[1] Nigg JT (2001) Is ADHD a disinhibitory disorder? Psychological Bulletin 127: 571. 11548968 10.1037/0033-2909.127.5.571

[2] Barkley RA (1997) Behavioral inhibition, sustained attention, and executive functions: Constructing a unifying theory of ADHD. Psychological Bulletin 121: 65–94. 9000892 10.1037/0033-2909.121.1.65

[3] American Psychiatric Association (2000) Diagnostic and Statistical Manual of Mental Disorders - Text Revision. Washington DC: The American Psychiatric Association.

[4] Ebejer JL, Coventry WL, Byrne B, Willcutt EG, Olson RK, et al (2010) Genetic and Environmental Influences on Inattention, Hyperactivity-Impulsivity, and Reading: Kindergarten to Grade 2. Scientific Studies of Reading 14: 293–316. 20823940 10.1080/10888430903150642 PMC2930267

[5] Derks EM, Dolan CV, Hudziak JJ, Neale MC, Boomsma DI (2007) Assessment and etiology of attention deficit hyperactivity disorder and oppositional defiant disorder in boys and girls. Behavior Genetics 37: 559–566. 17443404 10.1007/s10519-007-9153-4 PMC1914288

[6] Boomsma DI, Saviouk V, Hottenga J-J, Distel MA, de Moor MHM, et al (2010) Genetic Epidemiology of Attention Deficit Hyperactivity Disorder (ADHD Index) in Adults. PLoS ONE 5: e10621. 20485550 10.1371/journal.pone.0010621 PMC2868902

[7] Thapar A, Langley K, Asherson P, Gill M (2007) Gene-Enivornment interplay in attention-deficit hyperactivity disorder and the importance of a developmental perspective. British Journal of Psychiatry 190: 1–3. 17197648 10.1192/bjp.bp.106.027003

[8] Mick E, Biederman J, Prince J, Fischer MJ, Faraone SV (2002) Impact of low birth weight on attention-deficit hyperactivity disorder. Journal of Developmental and Behavioral Pediatrics 23: 16–22. 11889347 10.1097/00004703-200202000-00004

[9] Asbury K, Dunn J, Pike A, Plomin R (2003) Nonshared environmental influences on individual differences in early behavioral development: A monozygotic twin differences study. Child Development 74: 933–943. 12795399 10.1111/1467-8624.00577

[10] Banerjee T, Middleton F, Faraone S (2007) Environmental risk factors for attention-deficit hyperactivity disorder. Acta Paediatrica 96: 1269–1274. 17718779 10.1111/j.1651-2227.2007.00430.x

[11] Johnston C, Mash EJ (2001) Families of children with attention-deficit/hyperactivity disorder: review and recommendations for future research. Clinical Child and Family Psychology Review 4: 183–207. 11783738 10.1023/a:1017592030434

[12] Harpin V (2005) The effect of ADHD on the life of an individual, their family, and community from preschool to adult life. British Medical Journal 90: i2. 10.1136/adc.2004.059006 PMC1765272 15665153

[13] Willcutt EG, Pennington BF (2000) Psychiatric comorbidity in children and adolescents with reading disability. Journal of Child Psychology and Psychiatry 41: 1039–1048. 11099120

[14] Fliers E, Vermeulen S, Rijsdijk F, Altink M, Buschgens C, et al (2009) ADHD and poor motor performance from a family genetic perspective. Journal of the American Academy of Child & Adolescent Psychiatry 48: 25–34. 19218895 10.1097/CHI.0b013e31818b1ca2

[15] Kats-Gold I, Priel B (2009) Emotion, understanding, and social skills among boys at risk of attention deficit hyperactivity disorder. Psychology in the Schools 46: 658–678.

[16] Busch B, Biederman J, Cohen LG, Sayer JM, Monuteaux MC, et al (2002) Correlates of ADHD among children in pediatric and psychiatric clinics. Psychiatric Services 53: 1103–1111. 12221308 10.1176/appi.ps.53.9.1103

[17] Das D, Cherbuin N, Butterworth P, Anstey KJ, Easteal S (2012) A Population-Based Study of Attention Deficit/Hyperactivity Disorder Symptoms and Associated Impairment in Middle-Aged Adults. PloS one 7: e31500. 22347487 10.1371/journal.pone.0031500 PMC3275565

[18] De Graaf R, Kessler R, Fayyad J, Ten Have M, Alonso J, et al (2008) The prevalence and effects of adult attention-deficit/hyperactivity disorder (ADHD) on the performance of workers: results from the WHO World Mental Health Survey Initiative. Occupational and environmental medicine 65: 835. 18505771 10.1136/oem.2007.038448 PMC2665789

[19] Kessler R, Adler L, Ames M, Barkley R, Birnbaum H, et al (2005) The prevalence and effects of adult attention deficit/hyperactivity disorder on work performance in a nationally representative sample of workers. Journal of Occupational and Environmental Medicine 47: 565. 15951716 10.1097/01.jom.0000166863.33541.39

[20] Fayyad J, De Graaf R, Kessler R, Alonso J, Angermeyer M, et al (2007) Cross-national prevalence and correlates of adult attention-deficit hyperactivity disorder. The British Journal of Psychiatry 190: 402. 17470954 10.1192/bjp.bp.106.034389

[21] Kessler R, Adler L, Barkley R, Biederman J, Conners C, et al (2006) The prevalence and correlates of adult ADHD in the United States: results from the National Comorbidity Survey Replication. American Journal of Psychiatry 163: 716. 16585449 10.1176/appi.ajp.163.4.716 PMC2859678

[22] Barkley RA, Murphy KR (2006) Identifying new symptoms for diagnosing ADHD in adulthood. The ADHD Report 14: 7–11.

[23] Kooij JJ, Buitelaar JK, van den Oord EJ, Furer JW, Th. Rijnders CA, et al (2005) Internal and external validity of attention-deficit hyperactivity disorder in a population-based sample of adults. Psychological Medicine 35: 817–827. 15997602 10.1017/s003329170400337x

[24] Mannuzza S, Klein RG, Moulton JL (2003) Persistence of Attention-Deficit/Hyperactivity Disorder into adulthood: What have we learned from the prospective follow-up studies? Journal of Attention Disorders 7: 93. 15018358 10.1177/108705470300700203

[25] Biederman J, Mick E, Faraone SV (2000) Age-dependent decline of symptoms of attention deficit hyperactivity disorder: impact of remission definition and symptom type. American journal of psychiatry 157: 816–818. 10784477 10.1176/appi.ajp.157.5.816

[26] Polanczyk G, de Lima MS, Horta BL, Biederman J, Rohde LA (2007) The worldwide prevalence of ADHD: a systematic review and metaregression analysis. American journal of psychiatry 164: 942. 17541055 10.1176/ajp.2007.164.6.942

[27] Disney E, Elkins I, McGue M, Iacono W (1999) Effects of ADHD, conduct disorder, and gender on substance use and abuse in adolescence. American Journal of Psychiatry 156: 1515. 10518160 10.1176/ajp.156.10.1515

[28] Volk HE, Neuman RJ, Todd RD (2005) A Systematic Evaluation of ADHD and Comorbid Psychopathology in a Population-Based Twin Sample. Journal of the American Academy of Child & Adolescent Psychiatry 44: 768–775. 16034278 10.1097/01.chi.0000166173.72815.83

[29] Babinski LM, Hartsough CS, Lambert NM (1999) Childhood conduct problems, hyperactivity-impulsivity, and inattention as predictors of adult criminal activity. Journal of Child Psychology and Psychiatry 40: 347–347–355. 10190336

[30] Levy F, McLaughlin M, Wood C, Hay D, Waldman I (1996) Twin-sibling differences in parental reports of ADHD, speech, reading and behaviour problems. Journal of Child Psychology and Psychiatry 37: 569–578. 8807437 10.1111/j.1469-7610.1996.tb01443.x

[31] Bucholz KK, Cadoret R, Cloninger CR, Dinwiddie SH (1994) A new, semi-structured psychiatric interview for use in genetic linkage studies: A report on the reliability of the SSAGA. Journal of Studies on Alcohol 55: 159–158. 8189735 10.15288/jsa.1994.55.149

[32] Min SJ, Luke B, Gillespie B, Min L, Newman RB, et al (2000) Birth weight references for twins. American journal of obstetrics and gynecology 182: 1250–1257. 10819867 10.1067/mob.2000.104923

[33] Wears RL (2002) Advanced Statistics: Statistical Methods for Analyzing Cluster and Cluster randomized Data. Academic emergency medicine 9: 330–341. 11927463 10.1111/j.1553-2712.2002.tb01332.x

[34] Kessler RC, Adler LA, Barkley R, Biederman J, Conners CK, et al (2005) Patterns and predictors of attention-deficit/hyperactivity disorder persistence into adulthood: results from the national comorbidity survey replication. Biological psychiatry 57: 1442–1451. 15950019 10.1016/j.biopsych.2005.04.001 PMC2847347

[35] Kooij SJJ, Boonstra MA, Swinkels SHN, Bekker EM, de Noord I, et al (2008) Reliability, validity, and utility of instruments for self-report and informant report concerning symptoms of ADHD in adult patients. Journal of Attention Disorders 11: 445. 18083961 10.1177/1087054707299367

[36] Buschgens CJM, van Aken MAG, Swinkels SHN, Altink ME, Fliers EA, et al (1996) Differential family and peer environmental factors are related to severity and comorbidity in children with ADHD. Journal of neural transmission 115: 177–186. 10.1007/s00702-007-0838-x 18200433

[37] Acosta MT, Castellanos FX, Bolton KL, Balog JZ, Eagen P, et al (2008) Latent class subtyping of attention-deficit/hyperactivity disorder and comorbid conditions. Journal of the American Academy of Child & Adolescent Psychiatry 47: 797–807. 18520958 10.1097/CHI.0b013e318173f70b PMC2774844

[38] Conners CK, Erhardt D, Sparrow EP. Conner’s Adult ADHD Rating Scales: CAARS; 1999. MHS.

